# ICTV Virus Taxonomy Profile: *Herelleviridae*

**DOI:** 10.1099/jgv.0.001392

**Published:** 2020-02-05

**Authors:** Jakub Barylski, Andrew M. Kropinski, Nabil-Fareed Alikhan, Evelien M. Adriaenssens, ICTV Report Consortium

**Affiliations:** ^1^​ Department of Molecular Virology, Institute of Experimental Biology, Faculty of Biology, Adam Mickiewicz University in Poznan, Collegium Biologicum – Umultowska 89, 61-614 Poznan, Poland; ^2^​ Department of Pathobiology, University of Guelph, 50 Stone Road E, Guelph, Ontario N1G 2W1, Canada; ^3^​ Quadram Institute Bioscience, Norwich Research Park, Norwich NR4 7UQ, UK

**Keywords:** *Herelleviridae*, ICTV Report, taxonomy

## Abstract

Members of the family *Herelleviridae* are bacterial viruses infecting members of the phylum Firmicutes. The virions have myovirus morphology and virus genomes comprise a linear dsDNA of 125–170 kb. This is a summary of the International Committee on Taxonomy of Viruses (ICTV) Report on the family *Herelleviridae*, which is available at ictv.global/report/herelleviridae.

## Virion

**Fig. 1.**
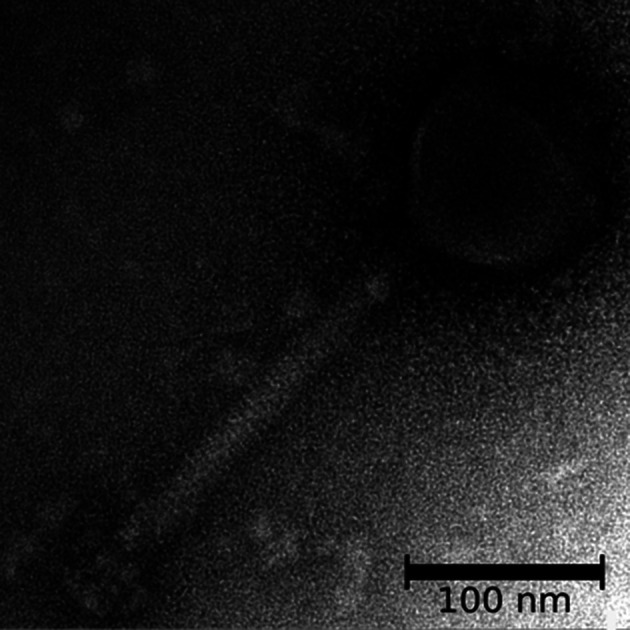
Transmission electron micrograph of Bacillus phage phiAGATE [8]. Virions were concentrated from bacteria-free lysates and stained with 2 % uranyl acetate (image, Jakub Barylski).

Virions have isometric, icosahedral heads 85–100 nm in diameter [1] (Table 1). The heads show clear capsomers, i.e. the subunits of the capsid are arranged in pentons and hexons that are assembled into the isometric, icosahedral capsid. The uncontracted tails are 130–185 nm in length. The tails have a baseplate of approximately 60 nm and a small collar (Fig. 1).

**Table 1.** Characteristics of members of the family *Herelleviridae*

Typical member:	Bacillus phage SPO1 (FJ230960), species *Bacillus virus SPO1*, genus *Okubovirus*
Virion	Head–tail morphology with contractile tail, heads generally isometric with diameters of 85–100 nm showing capsomers, uncontracted tails of 130–185 nm in length
Genome	Linear, terminally redundant, non-permuted dsDNA of 125–170 kbp
Replication	Phage-encoded DNA polymerase
Translation	Bacterial translation
Host range	Bacteria of the phylum Firmicutes
Taxonomy	Order *Caudovirales*, several subfamilies, >10 genera and >70 species

## Genome

**Fig. 2.**
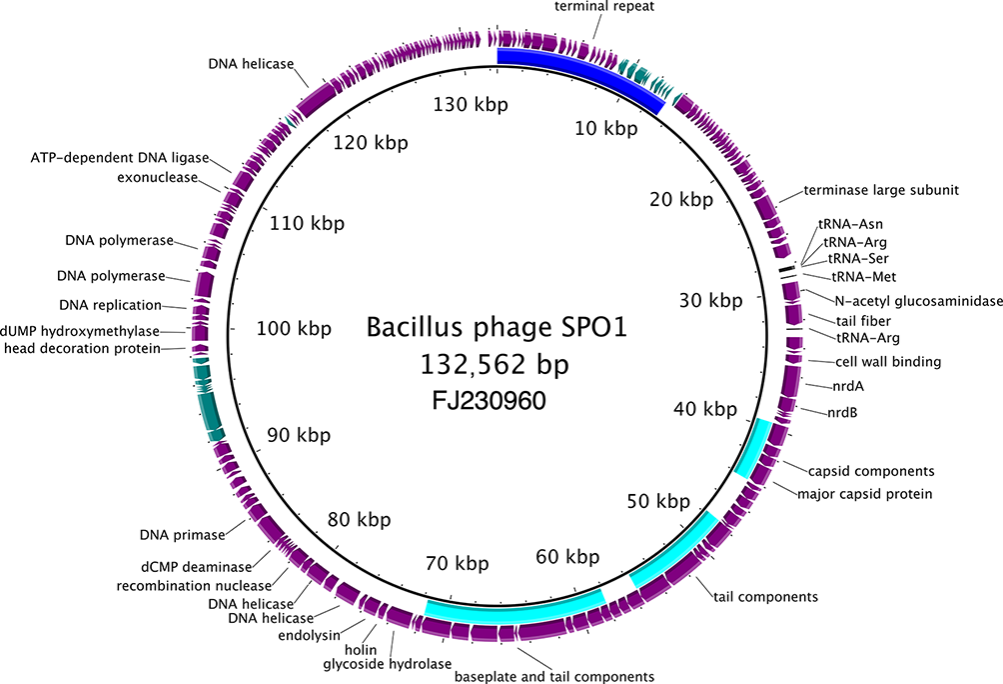
Genome organization of Bacillus phage SPO1. For convenience, the linear genome is shown as a circle with a single copy of the terminal repeat area indicated with a blue arc. Structural modules are indicated with cyan arcs. The predicted coding sequences on the plus strand are in purple, and those on the minus strand in teal. The five predicted tRNAs are indicated in black. Figure generated using BRIG [9].

Herelleviruses have linear genomes, the majority containing long, terminal repeats of various lengths [1, 2]. Genomes are of 125–170 kbp with 165–301 genes, including tRNA genes. Up to 24 tRNA codon specificities have been reported. In the Bacillus phage SPO1 genome, the majority of the coding sequences are in the same orientation; two islands of hypothetical coding sequences are transcribed from the opposite strand (Fig. 2). The terminal repeat of the Bacillus phage SPO1 genome contains a host-takeover module involved in phage propagation [3].

Core genes shared among all members of the family [4] comprise: DnaB-like helicase, baseplate J-like protein, tail sheath protein, terminase large subunit (intron-invaded), major capsid protein, prohead protease, portal protein, DNA primase, DNA polymerase I, RNA polymerase, recombination exonuclease, recombination endonuclease, tail tape measure protein and tail tube protein.

## Replication

Transcription is mediated by phage-encoded sigma factors to co-opt the host RNA polymerase [5]. Introns have been identified in a number of herellevirus genomes [6, 7]. Replication is mediated by a phage-encoded DNA polymerase.

## Taxonomy

The family *Herelleviridae* includes multiple subfamilies, each with one or more genera [4]. The subfamilies and genera are identified as well-supported monophyletic groups based on phylogenetic analysis of concatenated core gene markers and single core genes. Members of the same virus genus generally infect members of the same bacterial genus. Members of the same species are over 95 % identical in nucleotide sequence over the length of the genome, including the terminal repeat region.

## Resources

Current ICTV Report on the family *Herelleviridae*: ictv.global/report/herellevirdae. A detailed analysis of the phylogenetic and phylogenomic relationships in the family *Herelleviridae* has been published [4], with supplementary data in the Dryad repository at https://doi.org/10.5061/dryad.106q6g6.
